# NOVEL METHODS, MODELS, AND METRICS FOR MEDICARE DATA TO UNDERSTAND RECOVERY AFTER HOSPITALIZATION FOR INJURY

**DOI:** 10.1093/geroni/igae098.2083

**Published:** 2024-12-31

**Authors:** Michelle Shardell, Kristine Ensrud

**Affiliations:** Chair; Discussant

## Abstract

Medicare is the U.S. federal health insurance program for adults aged ≥65 years and some younger persons with specific conditions. The Medicare Minimum Data Set (MDS) is a standardized clinical assessment tool administered by the Centers for Medicare and Medicaid Services to facilitate care management for residents in Medicare and Medicaid certified nursing homes. Medicare administrative claims and MDS databases provide opportunity to generate novel and impactful real-world evidence, but deriving accurate conclusions can be difficult due to measurement and research design challenges. In this symposium, we illustrate four methods to overcome these challenges in the context of understanding mechanisms of post-hospitalization recovery among older adults, consistent with meeting theme “The Fortitude Factor”. First, we describe a new data science method to overcome unmeasured confounding when identifying factors associated with post-hospitalization monthly days at home (DAH), a person-centered metric that reflects aging in place, among older adults with Alzheimer’s Disease and Related Dementia (ADRD) who experience a hip fracture. We next examine the role of contextual socioeconomic disadvantage in recovery from hip fracture using weighting estimation to mitigate survival bias. To overcome bias from informative observation times in the MDS, we apply a flexible joint model to examine time-varying factors associated with post-fracture physical function among older adults with ADRD. Lastly, we innovatively apply latent variable analysis to identify post-hospitalization DAH trajectories among older adults with traumatic brain injury. We discuss how the gerontology community can use these bias-reducing approaches for policy-relevant health research with Medicare data.

